# Causal relationship between gut microbiota and diabetic nephropathy: a two-sample Mendelian randomization study

**DOI:** 10.3389/fimmu.2024.1332757

**Published:** 2024-03-08

**Authors:** Shuxiang Yan, Hua Wang, Baiyu Feng, Lin Ye, Anqun Chen

**Affiliations:** ^1^ Department of Nephrology, Hunan Key Laboratory of Kidney Disease and Blood Purification, Institute of Nephrology, The Second Xiangya Hospital at Central South University, Changsha, China; ^2^ Department of Orthopaedics, The Second Xiangya Hospital of Central South University, Changsha, Hunan, China; ^3^ Hunan Key Laboratory of Tumor Models and Individualized Medicine, The Second Xiangya Hospital of Central South University, Changsha, Hunan, China

**Keywords:** diabetic nephropathy, gut microbiota, type 2 diabetic mellitus, type 1 diabetic mellitus, Mendelian randomization, gut-kidney axis

## Abstract

**Objective:**

Emerging evidence has provided compelling evidence linking gut microbiota (GM) and diabetic nephropathy (DN) via the “gut-kidney” axis. But the causal relationship between them hasn’t been clarified yet. We perform a Two-Sample Mendelian randomization (MR) analysis to reveal the causal connection with GM and the development of DN, type 1 diabetes nephropathy (T1DN), type 2 diabetes nephropathy (T2DN), type 1 diabetes mellitus (T1DM), and type 2 diabetes mellitus (T2DM).

**Methods:**

We used summary data from MiBioGen on 211 GM taxa in 18340 participants. Generalized MR analysis methods were conducted to estimate their causality on risk of DN, T1DN, T2DN, T1DM and T2DM from FinnGen. To ensure the reliability of the findings, a comprehensive set of sensitivity analyses were conducted to confirm the resilience and consistency of the results.

**Results:**

It was showed that Class *Verrucomicrobiae* [odds ratio (OR) =1.5651, 95%CI:1.1810-2.0742,*P*FDR=0.0018], Order *Verrucomicrobiales* (OR=1.5651, 95%CI: 1.1810-2.0742, *P*FDR=0.0018) and Family *Verrucomicrobiaceae* (OR=1.3956, 95%CI:1.0336-1.8844, *P*FDR=0.0296) had significant risk of DN. Our analysis found significant associations between GM and T2DN, including Class *Verrucomimicrobiae* (OR=1.8227, 95% CI: 1.2414-2.6763, PFDR=0.0139), Order *Verrucomimicrobiae* (OR=1.5651, 95% CI: 1.8227-2.6764, PFDR=0.0024), *Rhodospirillales* (OR=1.8226, 95% CI: 1.2412-2.6763, PFDR=0.0026), and Family *Verrucomicroniaceae* (OR=1.8226, 95% CI: 1.2412-2.6763, PFDR=0.0083). The *Eubacteriumprotogenes* (OR=0.4076, 95% CI: 0.2415-0.6882, PFDR=0.0021) exhibited a protection against T1DN. Sensitivity analyses confirmed that there was no significant heterogeneity and pleiotropy.

**Conclusions:**

At the gene prediction level, we identified the specific GM that is causally linked to DN in both T1DM and T2DM patients. Moreover, we identified distinct microbial changes in T1DN that differed from those seen in T2DN, offering valuable insights into GM signatures associated with subtype of nephropathy.

## Introduction

One of the prevalent microvascular complications associated with diabetes is diabetic nephropathy(DN), usually diagnosed through symptoms such as albuminuria or a decreased estimated glomerular filtration rate (eGFR) (1, 2). Diabetes poses a significant global public health challenge. In modern times, diabetes has emerged as a highly consequential and prevalent chronic ailment, leading to severe and costly complications that pose a threat to life and well-being, as well as diminishing life expectancy (3). Approximately 537 million adults globally are affected by diabetes, with type 2 diabetes mellitus (T2DM) constituting almost 90% of the total cases, and the projected increase is expected to reach 783 million by the year 2045 (4). Of all diabetic patients diagnosed annually, 30% to 40% subsequently develop DN, and one-third of those individuals eventually advance to the end stage renal disease (ESRD) (5, 6). When comparing diabetic patients with kidney disease to those without, it is observed that the mortality rate of patients with DN is 30 times higher (7). T2DM is often associated with diabetic nephropathy (8), and it is crucial to note that as the incidence of T2DM increases, so does the frequency of DN (9). In recent decades, significant progress has been achieved in gaining a deeper understanding of the critical pathogenic aspects of DN, with the aim of developing enhanced therapeutic and preventive measures (10, 11). Despite these, current multifaceted intervention strategies intended to mitigate the risk of microangiopathy in people with diabetes have proven inadequate, primarily due to the lack of treatment options that can effectively and specifically address the molecular characteristics of DN. Hence, it is crucial and urgent to clarify the mechanism behind renal fibrosis in DN and to identify new biomarkers or targets associated with the gradual decline of renal function in patients with DN. Additionally, exploring the factors that impact nephropathy in patients with T2DM is a crucial measure, towards comprehending the disease’s impact and establishing research priorities.

Both physiology and disease state are significantly influenced by the gut microbiota, with connections to various health problems In recent times, there has been growing evidence suggesting a link between gut dysbiosis and diabetic nephropathy (DN), along with other conditions including diabetes, aging, obesity, and cancer (12–16). The latest research suggests that the onset and advancement of DN are linked to an altered gut microbial ecology or dysbiosis (17, 18). In age- and gender-matched patients with DN, lower levels of *Prevotella*_9 in the intestine were found compared to diabetic patients without kidney disease (19), capable of producing short-chain fatty acids and reducing the inflammatory response of kidney injury. The alteration of the intestinal microbiota is intricately linked to the progression of diabetes, as indicated by numerous studies. For instance, *Bacteroides fragilis, Akkermansia muciniphila* and *Roseburia intestinalis* have demonstrated the ability to enhance glucose metabolism and insulin sensitivity while also suppressing pro-inflammatory cytokines (20). Metabolic factors associated with oxidative stress and inflammatory response have been found to be interconnected with intestinal dysbiosis and T2DM, thereby impacting the onset and progression of diabetes-related complications (17, 21). However, the precise causal role of GM in the advancement of DN remains somewhat uncertain.

Mendelian randomization (MR) is a powerful methodology that utilizes summary data derived from genome-wide association studies (GWAS) to investigate potential causality of exposure factors and outcomes. The objective is to minimize the influence of confounding factors. The approach allows for a more robust analysis of the possible association between these factors, providing valuable insights into the underlying biological mechanisms implicated in the progression of diseases. The utilization of MR analysis is frequently employed as a means of assessing the potential correlation between exposure factors and outcomes (22). Recent studies utilizing MR analysis have made significant advancements in unveiling the causality of autoimmune disorders and GM (23) along with neuropsychiatric conditions (24).Nonetheless, the utilization of MR analysis methods to investigate the progression and pathogenic mechanism of DN remains unexplored.

This study places significant emphasis on investigating the causal relationship between exposure to GM and the outcome of DN, employing a method rooted in mendelian randomization analysis. The identification of specific GM strains correlated with DN patients offers prospects for the discovery of novel biomarkers, diagnostic, and treatment methods. Thus, this initiative could potentially be of significant benefit towards the development of precision medicine.

## Materials and methods

### General outline of MR analysis and three assumptions

**Figure 1:**
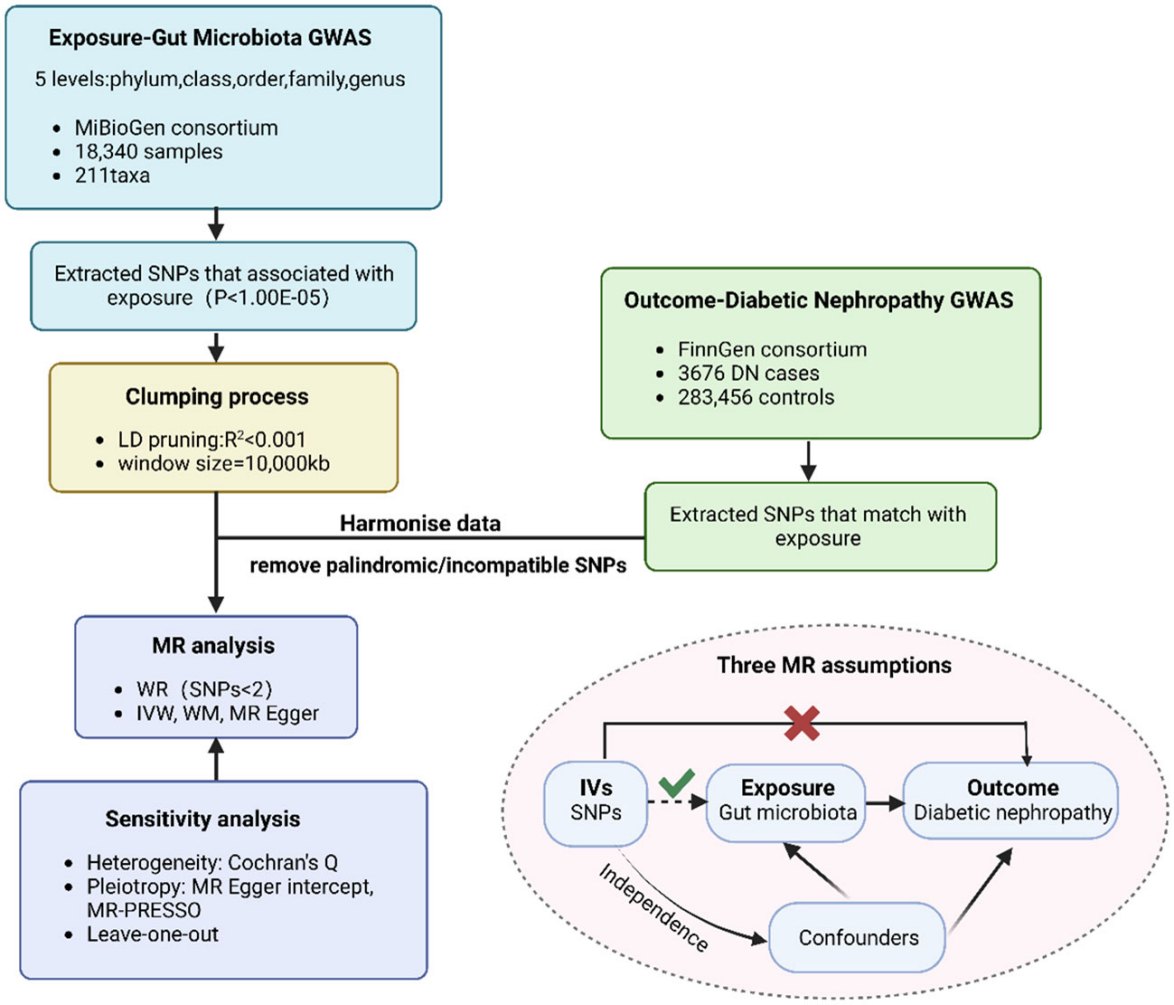
The flowchart of MR analysis. MR, Mendelian randomization n; GWAS, genome-wide association study; SNP, Single Nucleotide Polymorphism.

Overall, the causality of gut microbiota and diabetic nephropathy was examined via conducting a Two-Sample MR analysis. We assessed open GWAS summary statistics for DN as well as GM, and the workflow for this study between GM taxa and DN is presented in **Figure 1**. The MR analysis relied on three fundamental assumptions, as depicted in **Figure 1**: 1) the instrumental variables (IVs) which were screened for the analysis needed to be highly associated with exposure factors; 2) both of confounding factors and instrumental variables that affect GM taxa and DN should be independent of one another; 3) there was no evidence of horizontal pleiotropy, indicating that instrumental variables merely impacted DN via GM taxa. Furthermore, we incorporated GWAS data pertaining to T1DM, T2DM, as well as type 1 diabetic nephropathy (T1DN) and type 2 diabetic nephropathy (T2DN) outcomes. These data also satisfied the aforementioned three essential assumptions during the implementation of MR analysis.

### Data sources for exposure and outcome

We obtained the GWAS summary statistics of GM from the MiBioGen Consortium, a comprehensive multi-ethnic GWAS meta-analysis comprising 18,340 individuals from 24 cohorts. The data was accessed on January 12, 2023, from the consortium’s website (www.mibiogen.org). The summary statistics of GWAS typically included data on 211 taxonomic groups at various levels, ranging from genus to phylum. These groups encompassed 131 genera, 35 families, 20 orders, 16 classes, 9 phyla, and 122,110 related single nucleotide polymorphisms (SNPs) (25). The microbial composition was subtly profiled by targeting three different variable regions of the 16S rRNA gene. IVs of GM taxa were extracted at distinct 5 levels from this large-scale GWAS. Notably, the identification of these SNPs was limited to the European population, achieving a significance level (p<1e-05).

As for diabetic nephropathy, the statistics were extracted primarily from worldwide study in Europe (FinnGen (26), https://r8.finngen.fi/accessed on 12 January 2023) including 3676 DN cases and 283456 age/gender-matched healthy controls. We also acquired GWAS summary statistics from the FinnGen database (accessed on 12 January 2023), encompassing four outcomes: T1DM (8026 T1DM cases and 283320 healthy controls), T2DM (33043 T2DM cases and 284971 healthy controls), T1DN (1441 T1DN cases and 283224 healthy controls), and T2DN (2394 T2DN cases and 283224 healthy controls). According to ICD-10 standard (code: N08.3 *), when patients with diabetes mellitus has glomerular disorders, diabetes nephropathy could be defined as outcome. T1DM and T2DM can be classified based on the presence of insulin dependence in patients with DM, using the ICD-9/10 criteria (code: E10/E11*,250/250.A*). According to ICD-9/10 standard (code: N08.3 *), T1DN and T2DN were defined as outcome whether there was insulin dependence in the patients with diabetic nephropathy.

### Quality control of instrumental variables

Firstly, the SNPs that attained a p-value below the locus-wide significance threshold (p<1e-05) were chosen for ensuring the inclusion of appropriate IVs (27, 28). Secondly, we performed a linkage disequilibrium (LD) analysis (r²<0.001, clumping distance=10000kb) to assess the independence of these variables and the presence of LD effect. The palindromic and incompatible SNPs were further removed from the IVs. Additionally, we have excluded IVs with F-statistics below 10. The F-statistic is calculated using the formula F = beta^2^/se^2^, where beta represents the effect of SNP on the exposure, and se denotes the standard error of beta (29). Moreover, those instrumental variables that were not bound up with outcome were excluded if the p-value of the outcome variables was less than 0.05. Proxy SNP whose LD score was higher than 0.8 was utilized when there were SNPs that are missing in the outcome. R²(formula: 2 × EAF × (1-EAF) × beta^2^, where EAF represents the effect allele frequency of the SNP) was calculated to make sure the magnitude of the correlation of exposure and IVs (30). Phenoscanner V2 (31) was utilized to identify the possible confounders (BMI, blood pressure, blood lipids, heart disease, hypertension, etc.) that may be associated with the IVs. SNPs related to any of these potential confounders were excluded at the genome-wide significance level to prevent their interference with the effect of exposures on the outcome. Ensuring the rigor and reliability of our research findings necessitates the implementation of quality control measures for instrumental variables.

### Mendelian randomization analyses

The inverse variance weighted (IVW) analysis was employed as the primary statistical method for GM taxa that encompassed multiple SNPs. The Wald ratio (WR) method was employed to analyze GM taxa that consisted of a single SNP. Additionally, in order to provide further confirmation of the IVW result, we employed additional statistical methods such as MR-Egger regression and weighted median (WM) analysis as complementary approaches. The IVW method has the capability to integrate the Wald estimation of individual gene variants within a meta-analysis framework. When the horizontal pleiotropy is appropriately balanced, this method can yield unbiased results (32). IVW is commonly favored due to its ability to provide unbiased estimates of the status, while mitigating the impact of horizontal pleiotropy. The representation of the effect size can be achieved by utilizing the odds ratio in conjunction with a 95% confidence interval (CI). The effectiveness of MR Egger’s results was observed when the proportion of SNPs with pleiotropy exceeded 50% (33).The results from WM were regarded as the significant causal effect values if the number of SNPs with heterogeneity was over 50% (34). In cases where there was a discrepancy between the results obtained from different methods, the IVW method was chosen as the primary outcome.

Furthermore, we conducted a variety of sensitivity analyses to assess the robustness of the identified causal relationships. These analyses included the MR-Egger intercept test, as well as mendelian randomization pleiotropy residual sum and outlier (MR-PRESSO) analysis. Cochrane’s Q test was conducted to assess the heterogeneity among different associated with IVs. When the p-value of heterogeneity is less than 0.05, the random-effects IVW test was conducted to provide a more conservative yet robust estimate. When the p-value exceeded 0.05, it indicated that the observed outcome did not exhibit significant horizontal pleiotropy. MR-PRESSO (35) as the capability to assess and eliminate outliers exhibiting horizontal pleiotropy (p<0.05), thereby providing a refined causal estimate. We performed a leave-one-out analysis to determine whether the significant outcome was influenced by a single SNP and to assess the presence of outliers, as well as the stability of the results.

### Ethics statement

The GWAS statistics utilized in this study were readily accessible to the public for download. Approval from the relevant institutions had been obtained for all GWAS included in this study, indicating that ethical protocols had been adhered to. Therefore, no additional ethical approvals were necessary for this study.

### Statistical analysis

We performed a comprehensive analysis using MR analysis and various sensitivity analyses to assess the causal impact of gut microbiota on the development of diabetic nephropathy. All of the aforementioned analyses were conducted using the open-source packages TwoSampleMR (version 0.5.6) (36) as well as MR-PRESSO (version 1.0) in R (version 4.2.1, https://www.rproject.org/, accessed on 15 July 2022). The statistical significance of the estimates for the MR effect was assessed by applying a false discovery rate (FDR) threshold of less than 5%. This threshold was employed to correct for multiple testing. In addition, we employed the Bonferroni correction method to obtain a more stringent validation of the significantly causal relationship, taking into account the number of genera, families, orders, classes, and phyla under each level. The significance threshold was adjusted as follows: for genera, the adjusted p-value was 0.05 divided by 131 (3.81e-4); for families, it was 0.05 divided by 35 (1.4e-3); for orders, it was 0.05 divided by 20 (2.5e-3); for classes, it was 0.05 divided by 16 (3.1e-3); and for phyla, it was 0.05 divided by 9 (5.5e-3). Additionally, any p-value falling between 0.05 and the Bonferroni-corrected p-value was considered nominally significant. The project of our study (37) was guided by referencing the STROBE-MR guideline.

### Reverse Mendelian randomization analysis

To examine the potential causal effect of DN on the significant GM, a reverse Mendelian randomization analysis was performed. This analysis utilized single SNPs associated with DN as instrumental variables, with DN as the exposure and the identified causal GM as the outcome. The instrumental variable weighted (IVW), MR-Egger regression, weighted median, along with MR-PRESSO test methods were implemented via utilizing the TwoSampleMR package (version 0.5.6) as well as MR-PRESSO as a supplement (version 1.0) in R (version 4.2.1, accessed on 15 July 2022).

## Results

### The selection of instrumental variables

A total of 14,587 instrumental variables based on the MiBioGen consortium were initially found to achieve locus-wide significance (p<1e-5), but after removing the effects of both linkage disequilibrium and palindromic for specific flora, only 1043 instrumental variables remained. These taxa represented 9 phylum (87 SNPs), 16 class (132 SNPs),20 order (154 SNPs), 35 family (221 SNPs) and 125 genera (449 SNPs) with each SNP showing adequate validity (all F>10) as evidenced in **Table 1**. Additionally, we ultimately included 1025 instrumental variables (**Supplementary Table S1**) which were selected from 211 flora in our analysis after removing those that might have been related to confounding factors of outcomes (n=18). The procedure for screening SNPs for the remaining four outcomes, which are T1DM, T2DM, T1DN, and T2DN, remains unchanged from the above-mentioned process. For detailed information, kindly consult the **Supplementary Materials Table S5-S8** with each SNP showing adequate validity (all F>10).

**Table 1:** Selection of IVs after quality control.

Taxonomies	Taxa	NSNP	Palindromic	IVs
Genus	125	524	75	449
Family	35	261	40	221
Order	20	184	30	154
Class	16	157	25	132
Phylum	9	99	12	87
Total	205	1225	182	1043

IV, Instrumental Variable; SNP, Single Nucleotide Polymorphism; NSNP is the number of SNPs being used as IVs.

### Class *Verrucomicrobiae*, order *Verrucomicrobiales*, and family *Verrucomicrobiaceae* are strongly associated with an increased risk of DN

**Figure 2:**
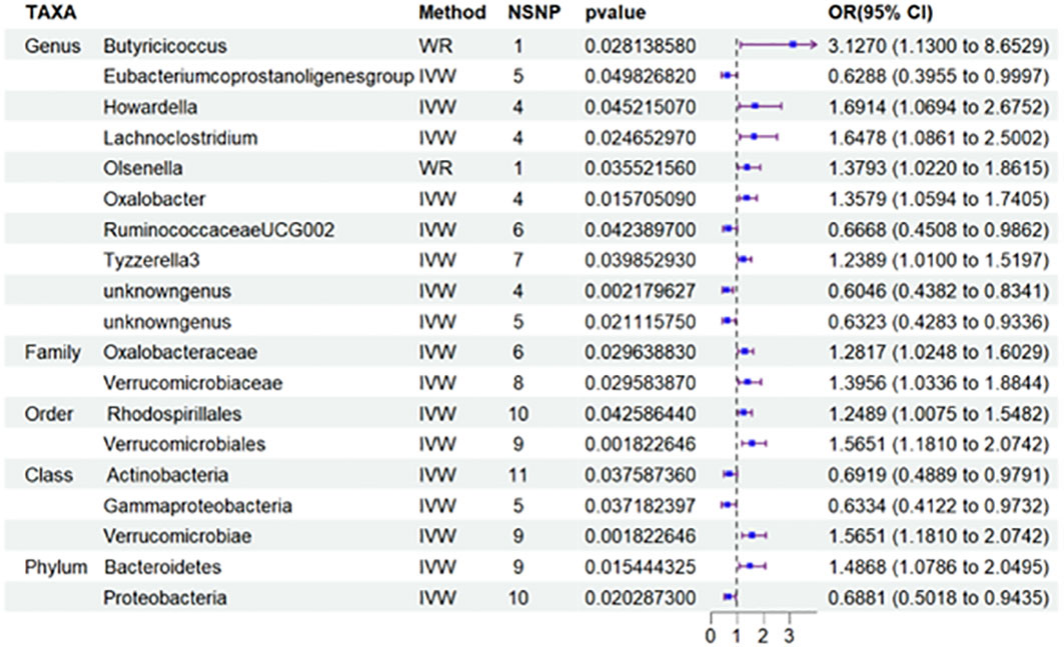
Results of MR Analysis between 19 potential casually microbiotas and DN. MR, Mendelian randomization; SNP, single-nucleotide polymorphism; IV, instrumental variable; IVW, inverse-variance weighted; WR,wald ratio; OR, odds ratio; CI, confidence interval. SNP, single nucleotide polymorphism; DN, diabetic nephropathy.

Nineteen causal relationships were identified through IVW results based on MR analysis. **Figure 2** depicts the association between potential causally linked bacterial taxa and diabetic nephropathy. Genus *Butyricicoccus*, Genus *Howardella*, Genus *Lachnoclostridium*, Genus *Oxalobacter*, Genus*Tyzzerella3*, Genus *unknowngenus*, Family *Oxalobacteraceae*, Family *Verrucomicrobiaceae*, Order *Rhodospirillales*, Order *Verrucomicrobiales*, Class *Verrucomicrobiae* and Phylum *Bacteroidetes* were associated with a higher risk of DN. While Genus *Eubacerium*, Genus *RuminococcaceaeUCG002*, Genus *unknowngenus*, Class *Actinobacteria*, Class *Gammaproteobacteria* and Phylum *Proteobacteria* were found to be linked with a decreased likelihood of developing DN. Sensitivity analyses were conducted using Cochrane’s Q test, MR-Egger as well as MR-PRESSO Global tests (**Supplementary Table S3**) to assess the presence of significant heterogeneity and pleiotropy. The results of these analyses confirmed that no significant heterogeneity or level of pleiotropy was observed.

**Figure 3:**
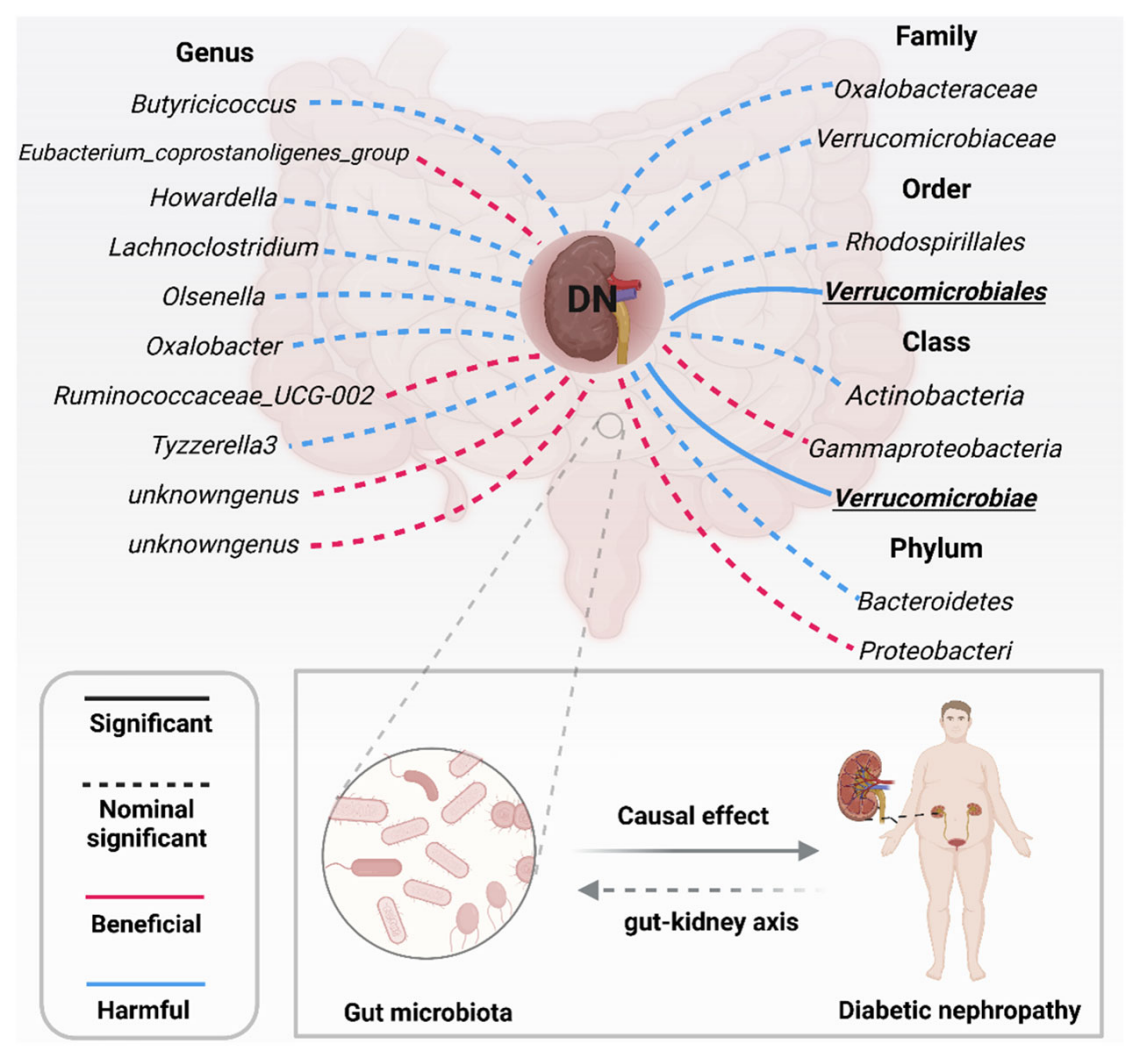
Significant and nominally significant links between GM and DN (*P* value corrected by the Bonferroni-corrected test). DN, diabetic nephropathy; Significant P was marked in bold.

More importantly, results from the FDR (**Table 2**) unveiled that a higher level of Class *Verrucomicrobiae*[odds ratio(OR)=1.5651,95%CI:1.1810-2.0742, PFDR=0.0018], Order *Verrucomicrobiales*(OR=1.5651,95%CI:1.1810-2.0742, PFDR=0.0018) and Family *Verrucomicrobiaceae* (OR=1.3956, 95%CI:1.0336-1.8844, PFDR=0.0296) retain a strong causal relationship with DN, which were also supported by the weighted median method (**Supplementary Table S4**). **Figure 3** (based on Bonferroni-corrected test) shows significant and nominally significant links between gut microbiota and diabetic nephropathy. Results from MR-Egger as well as MR-PRESSO tests (**Supplementary Table S3**) exhibited no indications of horizontal pleiotropy or outlier effects (p > 0.05). Additionally, findings derived from Cochrane’s Q test (**Supplementary Table S3**) did not show significant heterogeneity (p > 0.05). Furthermore, the leave one-out analysis revealed no significant difference in causal estimations of Class Verrucomicrobiae, Order Verrucomicrobiales and Family Verrucomicrobiaceae on diabetic nephropathy, unveiling that all of the causal associations which were distinguished by our study were not driven by specific single IV (**Supplementary Figure S3**).

**Table 2:** Casual effects of MR Analysis between GM and DN (*P* value corrected by FDR).

Outcome	Level	Exposure	Method	NO.SNP	*P*	*PFDR*
DN	genus	*Oxalobacter*	IVW	4	0.01570509	**0.026529**
			MR Egger		0.97126844	0.20911
			WM		0.12379308	1.640656
		*Lachnoclostridium*	IVW	4	0.02465297	**0.048915**
			MR Egger		0.90397821	1.793608
			WM		0.32419244	0.643239
		*Tyzzerella3*	IVW	7	0.03985293	**0.044479**
			MR Egger		0.98491188	1.099232
			WM		0.12705784	0.141806
	family	*Verrucomicrobiaceae*	IVW	8	0.02958387	**0.029584**
			MR Egger		0.56294025	0.56294
			WM		0.04102681	**0.041027**
	order	*Verrucomicrobiales*	IVW	9	0.001822646	**0.001823**
			MR Egger		0.498500366	0.4985
			WM		0.039104132	**0.039104**
	class	*Verrucomicrobiae*	IVW	9	0.001822646	**0.001823**
			MR Egger		0.498500366	0.4985
			WM		0.030097269	**0.030097**
	phylum	*Proteobacteria*	IVW	10	0.0202873	**0.026084**
			MR Egger		0.99720158	1.282116
			WM		0.09815853	0.126204

The bold values refer to the P-values still less than 0.05 after FDR correction.

### Class *Verrucomimicrobiae*, order *Verrucomimicrobiae*, *Rhodospirillales* and family *Verrucomicroniaceae* are highly correlated with risk of T2DN, while the genus *Eubacterium* might exhibit a protective effect against T1DN

**Figure 4:**
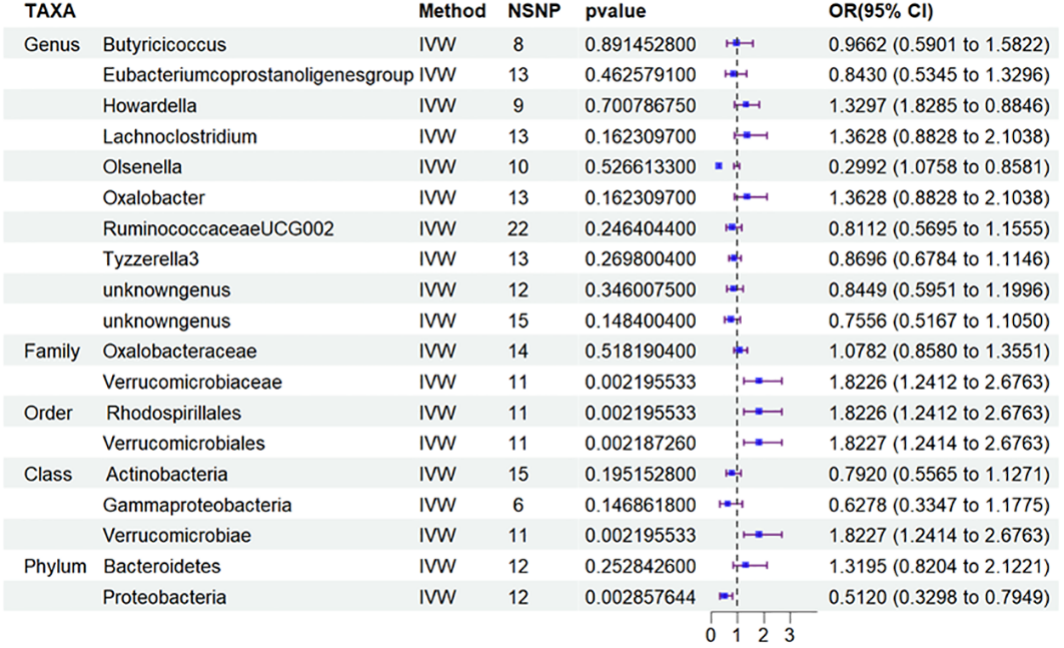
Results of MR Analysis between 19 potential casually microbiotas and T2DN. MR, Mendelian randomization; SNP, single-nucleotide polymorphism; IV, instrumental variable; IVW, inverse-variance weighted; WR,wald ratio; OR, odds ratio; CI, confidence interval; T2DN, type 2 diabetic nephropathy.

Besides, to investigate the potential correlation between the distribution of intestinal flora in DN and the specific type of diabetic nephropathy, particularly T2DN, we performed MR analysis (**Figure 4**) and applied FDR correction to 19 types of GM that were preliminarily identified as being associated with DN in the MR analysis. The results (**Table 3**) revealed that Class *Verrucomimicrobiae* (OR=1.8227, 95% CI: 1.2414-2.6763, PFDR=0.0139), Order *Verrucomimicrobiae* (OR=1.5651, 95% CI: 1.8227-2.6764, PFDR=0.0024), *Rhodospirillales* (OR=1.8226, 95% CI: 1.2412-2.6763, PFDR=0.0026), and Family *Verrucomicroniaceae* (OR=1.8226, 95% CI: 1.2412-2.6763, PFDR=0.0083) were negatively associated with T2DN.Conversely, the *Eubacterium protogenes group* (OR=0.4076, 95% CI: 0.2415-0.6882, PFDR=0.0021) exhibited a protective effect against T1DN.Clearly, the MR analysis outcomes for T2DN and DN exhibit a higher level of consistency.

**Table 3:** Casual effects of MR Analysis between GM and T1DN/T2DN (*P* value corrected by FDR).

Outcome	Level	Exposure	Method	NO.SNP	*P*	*PFDR*
T1DN	genus	*Eubacteriumcoprostanoligenesgroup*	IVW	13	0.000783	**0.002125763**
			MR Egger		0.120655	0.327492218
			WM		0.002616	**0.007100466**
T2DN	family	*Verrucomicrobiaceae*	IVW	11	0.002196	**0.008343025**
			MR Egger		0.709418	2.695786857
			WM		0.084151	0.319773891
	order	*Verrucomicrobiales*	IVW	11	0.002187	**0.002444585**
			MR Egger		0.706851	0.790009472
			WM		0.090536	0.101187786
		*Rhodospirillales*	IVW	11	0.002196	**0.002607195**
			MR Egger		0.082865	0.842433393
			WM		0.709418	0.098402182
	class	*Verrucomicrobiae*	IVW	11	0.002187	**0.013852647**
			MR Egger		0.706851	4.47672034
			WM		0.075537	0.478403913
	phylum	*Proteobacteria*	IVW	12	0.002858	**0.002857644**
			MR Egger		0.697337	0.697336558
			WM		0.047523	**0.047522824**

No. SNP is the number of SNPs being used as IVs; Significant P was marked in bold; MR, Mendelian randomization; SNP, single-nucleotide polymorphism; IV, instrumental variable; IVW, inverse-variance weighted; WM, weighted median; OR, odds ratio; CI, confidence interval; PFDR, P value corrected by false discovery rate (FDR). SNP: single nucleotide polymorphism; T2DN, type 2 diabetic nephropathy; T1DN, type 1 diabetic nephropathy. The bold values refer to the P-values still less than 0.05 after FDR correction.

**Figure 5:**
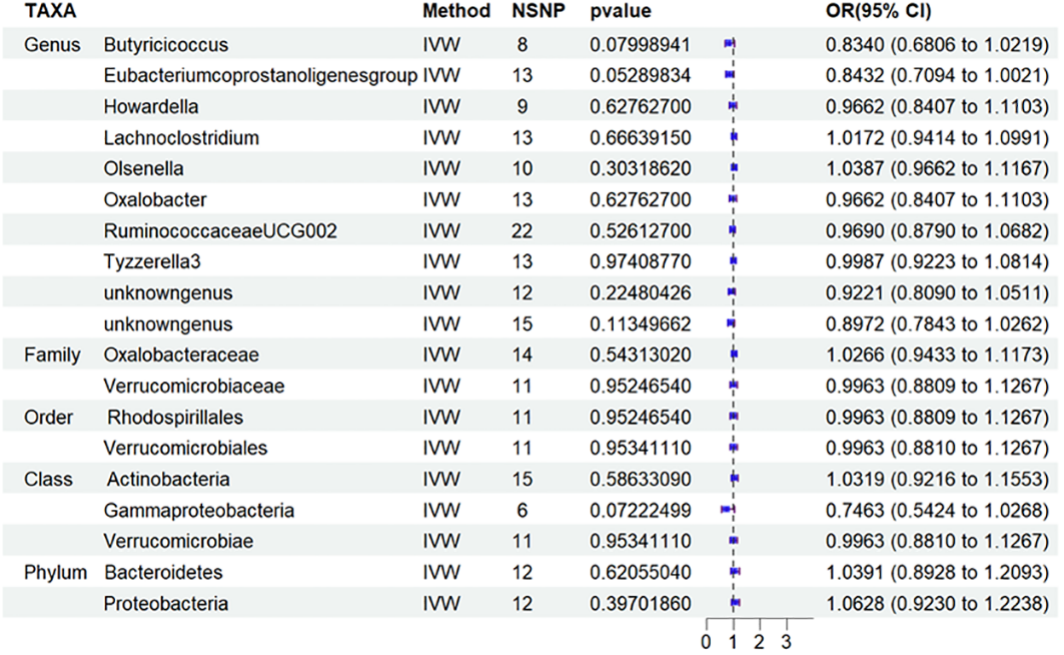
Results of MR Analysis between 19 potential casually microbiotas and T2DM MR, Mendelian randomization; SNP, single-nucleotide polymorphism; IV, instrumental variable; IVW, inverse-variance weighted; WR,wald ratio; OR, odds ratio; CI, confidence interval; T2DM, type 2 diabetic mellitus.

Based on this discovery, we proceeded to perform MR analysis on the aforementioned 19 types of GM and T2DM to investigate the variation in GM distribution across different stages of diabetic nephropathy. Additionally, we conducted an analysis on T1DM to aid in the verification process. However, based on the current results, we have not identified any significant findings in relation to T2DM (**Figure 5**) and T1DM.Sensitivity analyses of T1DN and T2DN were conducted using Cochrane’s Q test, MR-Egger, together with MR-PRESSO Global tests (**Table 4**), which collectively confirmed the absence of significant heterogeneity and pleiotropy. The **Supplementary Materials** contain the MR analysis (**Supplementary Tables S9-12**) and sensitivity analysis results (**Supplementary Tables S13-16**) for the remaining gut microbiota.

**Table 4:** Results of sensitivity analysis causal GM on DN/T1DN/T2DN.

Outcome	Level	Exposure	IVW_Q	IVW_Q_P	MR_Egger_Q	MR_Egger_Q_P	egger_intercept	pleiotropy_P	MR- PRESSO
DN	genus	*Oxalobacter*	1.430393	0.698426	1.267641	0.530561	0.045109	0.725679	0.7086667
		*Lachnoclostridium*	2.525022	0.470786	1.850822	0.396368	0.049963	0.497892	0.6943333
		*Tyzzerella3*	2.045882	0.915431	1.900832	0.86269	0.028881	0.718956	0.9226667
	family	*Verrucomicrobiaceae*	3.810355	0.801343	3.729566	0.713218	-0.020962	0.785785	0.8306667
	order	*Verrucomicrobiales*	8.096689	0.424083	7.994374	0.33309	-0.023595	0.773392	0.4643333
	class	*Verrucomicrobiae*	8.096689	0.424083	7.994374	0.33309	-0.023595	0.773392	0.4473333
	phylum	*Proteobacteria*	3.539969	0.939007	2.96262	0.936681	-0.024781	0.469151	0.95
T1DN	genus	*Eubacteriumcoprostanoligenesgroup*	8.989703	0.70381	8.2641	0.689469	0.054884	0.412473	0.7263333
T2DN	famliy	*Verrucomicrobiaceae*	5.111512	0.883605	4.799634	0.851413	0.028733	0.590152	0.9
	order	*Verrucomicrobiales*	5.104591	0.884083	4.796859	0.851645	0.02855	0.592592	0.91
	class	*Verrucomicrobiae*	5.104591	0.884083	4.796859	0.851645	0.02855	0.592592	0.905
	phylum	*Proteobacteria*	9.812906	0.547291	9.299518	0.503934	-0.029539	0.490071	0.5913333

### DN results in an increased occurrence of order *Rhodospirillales* and phylum *Proteobacteria* based on reverse analysis

Ulteriorly, we performed a reverse MR analysis, which show no clear evidence of reverse causality from DN to Class *Verrucomicrobiae*, Order *Verrucomicrobiales* and Family *Verrucomicrobiaceae* (**Supplementary Table S5**). Moreover, the reverse MR analysis revealed that DN may result in an increased occurrence of Order *Rhodospirillales* (β=0.0578, 95%CI:0.0129-0.1027 p=0.01161) and Phylum *Proteobacteria* (β=0.0402, 95%CI:0.0085-0.0719, p=0.01286) based on IVW results (**Supplementary Table S17**). The sensitivity analyses of the Cochrane’s Q test, MR-Egger as well as MR-PRESSO Global tests (**Supplementary Table S18**) indicated that there was no significant heterogeneity (p>0.05) or level of pleiotropy (p>0.05). No clear association was found for other gut microbiotas identified (p>0.05) except for the two. More detailed information on the reverse MR analyses was display in **Supplementary Table S17**.

## Discussion

The progress of research on the “gut-kidney” axis has faced obstacles due to several confounding factors, such as dietary habits. These factors have presented challenges in investigating the causality of GM and DN in a cross-sectional approach. To overcome these challenges, we utilized MR analysis and evaluated the causality between microflora and DN from point of host genetics. Our findings confirmed that GM taxa can modify susceptibility to DN, demonstrating the significant impact of gut microbiota on this condition. Total of 19 microflora that are associated with DN were identified in our research. We used two correction methods to correct the *P*-value to identify a stronger causal relationship among them. Class *Verrucomicrobiae* [odds ratio (OR)=1.5651, 95%CI: 1.1810-2.0742, *P*FDR=0.0018], Order *Verrucomicrobiales*(OR=1.5651, 95%CI:1.1810-2.0742, *P*FDR=0.0018) along with Family *Verrucomicrobiaceae*(OR=1.3956, 95%CI:1.0336-1.8844, *P*FDR=0.0296) still exert a higher risk on DN. Furthermore, the stricter Bonferroni-corrected test also provided the proof that *Verrucomicrobiae* and *Verrucomicrobiales* were strongly causally correlated with DN. The opposite causality analysis unlocked that DN support the augment of Order *Rhodospirillales* and Phylum *Proteobacteria*, suggesting that GM and DN may exist interaction. Collectively, our findings present innovative ideas that targeting regulation of dysbiosis in specific GM taxa could be a promising approach for DN prevention and therapy.

It is worth adding that to examine the potential relationship between the distribution of intestinal flora in DN and the specific type of diabetic nephropathy, specifically T2DN, we conducted MR analysis and employed FDR correction on 19 types of gut microbiota that were initially identified as being linked to DN in the aforementioned MR analysis. The results revealed that Class *Verrucomimicrobiae* (OR=1.8227, 95% CI: 1.2414-2.6763, PFDR=0.0139), Order *Verrucomimicrobiae* (OR=1.5651, 95% CI: 1.8227-2.6764, PFDR=0.0024), *Rhodospirillales* (OR=1.8226, 95% CI: 1.2412-2.6763, PFDR=0.0026), and Family *Verrucomicroniaceae* (OR=1.8226, 95% CI: 1.2412-2.6763, PFDR=0.0083) were negatively related to T2DN. Conversely, the *Eubacterium protogenes group* (OR=0.4076, 95% CI: 0.2415-0.6882, PFDR=0.0021) showed a protective effect on T1DN. It is evident that the MR analysis results for T2DN and DN demonstrate a higher degree of agreement. Building on this finding, we subsequently conducted MR analysis on the total of 19 microflora and T2DM to quest the variations in gut microbiota distribution across different stages of diabetic nephropathy. Furthermore, we carried out an analysis on T1DM to facilitate the verification process. However, based on the current findings, we have not discovered any significant results concerning T2DM and T1DM.

The gut microbiome in the digestive tract is often regarded as the “second genome of human” because of its significant role in regulating human health (38). It influences metabolic and immune functions through its metabolic activity, genes, and intermediaries. The gut microbiota is primarily made up of six major phyla: *Actinobacteria*, *Bacteroidetes*, *Fusobacteria*, *Firmicutes*, *Proteobacteria*, and *Verrucomicrobia*. The most commonly seen bacteria in the gut microbiota are *Bacteroidetes* as well as *Firmicutes*, constituting approximately 90% of the GM (39, 40). By studying the interaction between the gut microbiome and plasma metabolomics in an experimental model of DN in mice, evidence supporting the existence of the gut-kidney axis has been found. This study confirmed the involvement of gut microbiota and circulating metabolites in the progression of DN (41, 42). It is noteworthy that imbalanced gut microbiota is found in DN patients’ fecal samples where there is an increased presence of *Proteobacteria*, *Verrucomicrobia* and *Fusobacteria (* 43). Furthermore, one previous study (44) has indicated a significant decrease in Butyrate-producing microflora (such as *Clostridium*, *Eubacterium*, along with *Roseburia intestinalis*) and probiotics in the GM of individuals with type 2 diabetes and DN. In addition, several studies have demonstrated that specific metabolites originating from the GM play extremely important part of DN, such as lipopolysaccharide (LPS) (45), short-chain fatty acids (SCFAs) (46), and bile acids (BAs) (47). Interventions targeting the gut microbiota, such as supplementation with probiotics (48, 49) and administration of antibiotics (50), have been proven to partially improve both pathology and renal function of DN. Patients with DN exhibited dysbiosis in the composition, richness, and diversity of gut microbiota (19, 51, 52). However, the clear and solid mechanisms through which gut microbiota influences DN is still not completely clarified.

The findings indicate a significant causal correlation between the Order *Verrucomicrobiae* and Class *Verrucomicrobiales* with DN, both of which are categorized under the Phylum *Verrucomicrobia*. *Verrucomicrobia* is a recently identified bacterial phylum, encompassing a limited number of documented species, predominantly found in aquatic and terrestrial ecosystems, as well as in human fecal matter (53). Wu et al. (42) observed a downregulation of *Akkermansia*, a genus within the phylum *Verrucomicrobia*, in the colon of db/db mice compared to non-diabetic controls. While Wang et al. discovered that the mice with DN exhibited a significant increase in *Verrucomicrobiota* compared to the control mice and *Akkermansia* was found to be enriched in the DN mice (54). *Akkermansia* has been considered to be a beneficial microflora (55) capable of improving gut barrier function along with mitigating metabolic disorders like insulin resistance, obesity, as well as glucose intolerance (56). Whether the controversial result due to the different types of the diabetes need to be further investigated. Given that most renal complications associated with diabetes stem from type 2 diabetes, our initial focus was on conducting MR analysis involving above-mentioned 19 specific types of GM and T2DN. Surprisingly, after adjusting the P value, we stumbled upon a remarkable alignment between the results of T2DN and DN. This discovery underscores the importance of including patients with T2DM or animal models of T2DM in future studies investigating the correlation between DN and GM, as it has the potential to generate substantial insights.

Furthermore, a clinical study utilizing 16sRNA fecal analysis of patients with DN revealed an enrichment of *Verrucomicrobia* in comparison to healthy individuals (42, 43), suggesting its susceptibility to the progression of DN. Nevertheless, multiple researches have demonstrated that no discernible difference existed in the proportions of *Verrucomicrobia* between patients with DN and those with diabetes (19, 57–59). Interestingly, In the pre-diabetes (Pre-DM) cohort, Zhang et al. observed a notable reduction in the relative abundance of *Verrucomicrobia* and *Verrucomicrobiae*. This suggests the potential for *Verrucomicrobiae* to function as a signaling molecule or a diagnostic biomarker for the advancement of glucose intolerance, or serve as a beneficial microorganism to protect against type 2 diabetes (60). Consequently, it is evident that *Verrucomicrobia* experiences down-regulation in the initial phases of diabetes, and up-regulation in the nephrotic stage of diabetes. Nevertheless, there is a lack of research examining the dynamic changes of this microorganism across the early, middle, and late stages of diabetes. Therefore, our study delves further into genetic explanations. Nineteen types of GM related to DN were analyzed as the exposure, with diabetic mellitus as the outcome. The study aimed to ascertain whether there were any changes in the distribution of GM during different stages of diabetes. Neither the patients diagnosed with T1DM nor those diagnosed with T2DM exhibit significant alterations in the 19 types of microflorae. Nevertheless, it is important to note that these results do not necessarily imply the absence of significant variations in GM among DN during different time periods. However, our MR results also offer valuable insights and support. It was found that specific gut microbes demonstrate an elevated risk or a protective influence in T2DN and T1DN, whereas no alterations in these microbes were noted in the initial phases of T2DM and T1DM. This suggests that certain gut microbiota may not exert an influence during the initial phase of diabetes, but they may have a substantial effect in the later stages of diabetes, especially when combined with renal complications. Consequently, additional research is required to ascertain the potential impact of various disease stages on alterations in gut microbiota.

Although age, gender, dietary preferences, geographical location, and the use of antibiotics and probiotics are all known to have a significant impact on the GM in the composition (20, 61), reproducing the diversity and abundance of gut microbiota composition in different hosts with DN may present a considerable challenge. Our findings align with previous observational and functional studies, suggesting that *Verrucomicrobia* may be associated with an increased risk of DN. The existing mechanism may encompass various factors, such as the generation of metabolites by intestinal flora that can affect renal function, the control of inflammation and immune reactions, and the adjustment of intestinal barrier function. In a previous study, Salguero et al. (43) demonstrated a significantly higher abundance of *Verrucomicrobia* in DN mice compared to the controls, which was linked to increased levels of LPS. This can lead to systemic inflammation (62), activation, and overproduction of pro-inflammatory cytokines, including tumor necrosis factor-α (TNFα) and interleukin-6 (IL-6) (63). The *Verrucomicrobiaceae* family, a constituent of the *Verrucomicrobia* phylum, has exhibited a notable increase and was related to elevated levels of TNFα and interferon γ in the plasma of individuals diagnosed with Parkinson’s disease (64). Furthermore, the accumulation of urinary toxins and metabolic waste in patients with DN and severe renal impairment worsens the condition (65). Overall, through integrating evidence from MR analysis, functional studies, observational studies, and clinical trials, we have put forward the hypothesis that the impact of *Verrucomicrobia* on DN may vary depending on the specific species and strains involved. Despite this, the precise molecular mechanisms through which the GM contributes to the pathogenesis of DN remain incompletely understood. More investigation is needed to statement an association between *Verrucomicrobia* and DN. This research should aim to clarify the specific molecular mechanisms operating within the gut-kidney axis in DN, and to pinpoint potential therapeutic targets for the prevention and treatment of this disease.

Remarkably, a protective gut microbiota known as Eubacterium has been discovered in T1DN. The study findings indicate that children diagnosed with type 1 diabetes mellitus (T1DM) exhibit reduced levels of the *Blautiacoccoides-Eubacterium rectal group*, which is associated with butyrate production and the preservation of gut integrity, in comparison to their healthy counterparts (66). On the other hand, children who are in good health demonstrate elevated levels of butyrate-producing species like *Clostridium* IV and XIVa (66). Moreover, studies have shown that transplanting the gut microbiota of lean individuals to patients who have metabolic syndrome can induce substantial alterations in the gut microbiota. This leads to a heightened abundance of butyrate-producing intestinal flora, including *Eubacterium hallii*, which subsequently contributes to a notable enhancement in peripheral pancreatic insulin sensitivity six weeks post-transplantation (67). Additionally, in diseases like coronary artery disease, there is a notable decrease in butyrate-carrying intestinal flora, such as *Faecalibacterium*, *Roseburia*, and *Eubacterium rectum* (68). These findings are consistent with our conclusion in T1DN and call for further experimental research to validate it.

This study offers several advantages. Firstly, most of the current researches on the relationship between diabetes and gut microbes are derived from observational studies and it is vulnerable to interference from confounding factors. This is the first study utilizing MR analysis to offer a potential genetic mechanism. Therefore, our results are more robust and less prone to interference, resulting in increased stability. Secondly, the utilization of the most recent large Genome-Wide Association Studies (GWAS) enables the acquisition of genetic data from diverse sample populations and facilitates comprehensive analysis, thereby enhancing the robustness of results in comparison to smaller randomized controlled studies. Thirdly, previous meta-analysis studies have predominantly concentrated on examining the association between GM and DN at the phylum classification level. In contrast, our analysis advances this research by offering a more detailed comprehension of GM taxa and assessing the causal impact of each taxon on DN at the genus to phylum level. To the best of our knowledge, most of articles available solely focus on the outcomes of the disease itself. However, our research goes beyond the confines of diabetic nephropathy alone. We delve into a comprehensive analysis of various types of diabetes nephropathy as well as different forms of diabetes, thereby offering a wealth of genetic evidence.

It is crucial to give diplomatic recognition to the constraints of the research. Firstly, the microbiome represents an exposure phenotype that is only partially accounted for by genotype. This implies that precise calculation of the statistical powers in Mendelian randomization may not be entirely applicable. Secondly, given that the MR analysis is stemmed from three assumptions, additional experimental together with clinical verification studies are required to ascertain the clinical relevance of multiple microbial species. Thirdly, it is important to recognize that the use of FDR and the Bonferroni-corrected test could potentially lead to a false negative outcome. Following P-value correction, numerous correlations no longer demonstrated statistical significance, potentially attributable to the complex interaction between the intestinal and renal axes, which are commonly affected by virous distinct factors. Furthermore, even though two authors conducted independent bias checks, there remains the potential for subjective influences when utilizing Phenoscanner to eliminate confounding gene variables. Consequently, it is important to exercise prudence when interpreting the findings of the research.

In summary, our research utilized Mendelian randomization method to analyze and evaluate the causal relationship between intestinal microflora and diabetic nephropathy. The research findings revealed 19 nominal causalities and 2 robust causal associations. Notably, the Class *Verrucomicrobiae*, Order *Verrucomicrobiales* and Family *Verrucomicrobiaceae* were found to causally relate with a higher risk of DN in total and T2DN specifically. Furthermore, we have also unexpectedly discovered that Genus Eubacterium provides a protective effect specifically for T1DN, which was not observed in the T2DN. Our study has pinpointed specific microbiota using genetic prediction, which could serve as promising biomarkers for potential therapeutic targets of DN. Naturally, further experimental research is required in the future to substantiate and investigate potential gut microbiota targets and novel treatment possibilities for DN.

## Data availability statement

The original contributions presented in the study are included in the article/**Supplementary Material**. Further inquiries can be directed to the corresponding author.

## Author contributions

SY: Writing – original draft, Software, Methodology, Formal analysis, Data curation. HW: Writing – review & editing, Visualization, Data curation. BF: Writing – review & editing, Methodology, Data curation. LY: Writing – review & editing, Data curation. AC: Writing – review & editing, Project administration, Funding acquisition, Conceptualization.
