# Supplementary material for: Causal relationship between gut microbiota and diabetic nephropathy: a two-sample Mendelian randomization study
Source: Front Immunol. 2024 Mar 8;15:1332757. doi: 10.3389/fimmu.2024.1332757 (PMC10964483; doi:10.3389/fimmu.2024.1332757)

Supplementary Information

Supplement Table S17

Casual effects of MR Analysis between DN and causal GM (P<1.00E-05)

Exposure	Outcome	Method	NSNP	β	95%CI		P
	Class						
DN	*Gammaproteobacteria*	IVW	16	0.019101	-0.013464	0.051667	0.250292
	Verrucomicrobiae	IVW	15	0.035929	-0.002094	0.073952	0.064014
DN	Family						
	Verrucomicrobiaceae	IVW	15	0.035861	-0.002162	0.073884	0.06452
	Genus						
	Butyricicoccus	IVW	16	-8.23E-04	-0.033451	0.031804	0.960556
	Eubacteriumcoprostanoligenesgroup	IVW	16	-0.006155	-0.043457	0.031148	0.746405
	Howardella	IVW	12	0.019079	-0.051494	0.089651	0.596206
DN	*Lachnoclostridium*	IVW	16	-0.011066	-0.047813	0.02568	0.555023
	Olsenella	IVW	12	-0.004858	-0.08008	0.070364	0.899275
	Oxalobacter	IVW	12	0.019993	-0.044556	0.084541	0.543804
	RuminococcaceaeUCG002	IVW	16	0.028739	-0.002835	0.060313	0.074417
	Tyzzerella3	IVW	13	0.021698	-0.037667	0.081063	0.473758
	Order						
DN	***Rhodospirillales***	**IVW**	**15**	**0.057802**	**0.012913**	**0.102692**	**0.01161**
	Verrucomicrobiales	IVW	15	0.035671	-0.002382	0.073724	0.066163
	Phylum						
DN	*Bacteroidetes*	IVW	16	0.009145	-0.021315	0.039606	0.556225
	Proteobacteria	**IVW**	**16**	**0.040189**	**0.008524**	**0.071855**	**0.01286**

Supplement Table S18

Results of sensitivity analysis DN on causal GM (IVW: P<0.05)

Exposure	Outcome	IVW_Q	IVW_Q_P	Egger_intercept	Pleiotropy_P	MR-PRESSO
DN	Order					
	Rhodospirillales	10.87189	0.6960694	0.006074415	0.5784344	0.4486667
DN	Phylum					
	Proteobacteria	16.1341	0.3731958	0.000513166	0.9486579	0.7693333

Supplement Table S19

Characterization of Exposure and Outcome

Level	Name	Population	Reference or Website	Databese
Exposure	Gut Microbiota	European	Kurilshikov A, Medina-Gomez C, Bacigalupe R, et al. Large-scale association analyses identify host factors influencing human gut microbiome composition.	MiBioGen Consortium
Outcome	DN	European	https://risteys.finregistry.fi/endpoints/DM_NEPHROPATHY_	FinnGen
	T1DM	European	https://risteys.finregistry.fi/endpoints/T1D_WIDE	
	T2DM	European	https://risteys.finregistry.fi/endpoints/T2D_WIDE_	
	T1DN	European	https://risteys.finregistry.fi/endpoints/E4_DM1REN	
	T2DN	European	https://risteys.finregistry.fi/endpoints/E4_DM2REN	

Supplement Figure S1

Scatter plots of significant and nominal significant from GM on DN.(A:genus.Eubacerium;B:genus.Howardella;C:genus.Lachnoclostridium;D:genus.Oxalobacter;E:genus.RuminococcaceaeUCG002;F:genus.Tyzzerella3;G:genus.unknowngenus;H:genus.unknowngenus;I:family.Oxalobacteraceae;J:family.Verrucomicrobiaceae;K:order.Rhodospirillales;L:order.Verrucomicrobiales;M:class.Actinobacteria;N:class.Gammaproteobacteria;O:class.Verrucomicrobiae;P:phylum.Bacteroidetes;Q:phylum.Proteobacteria)

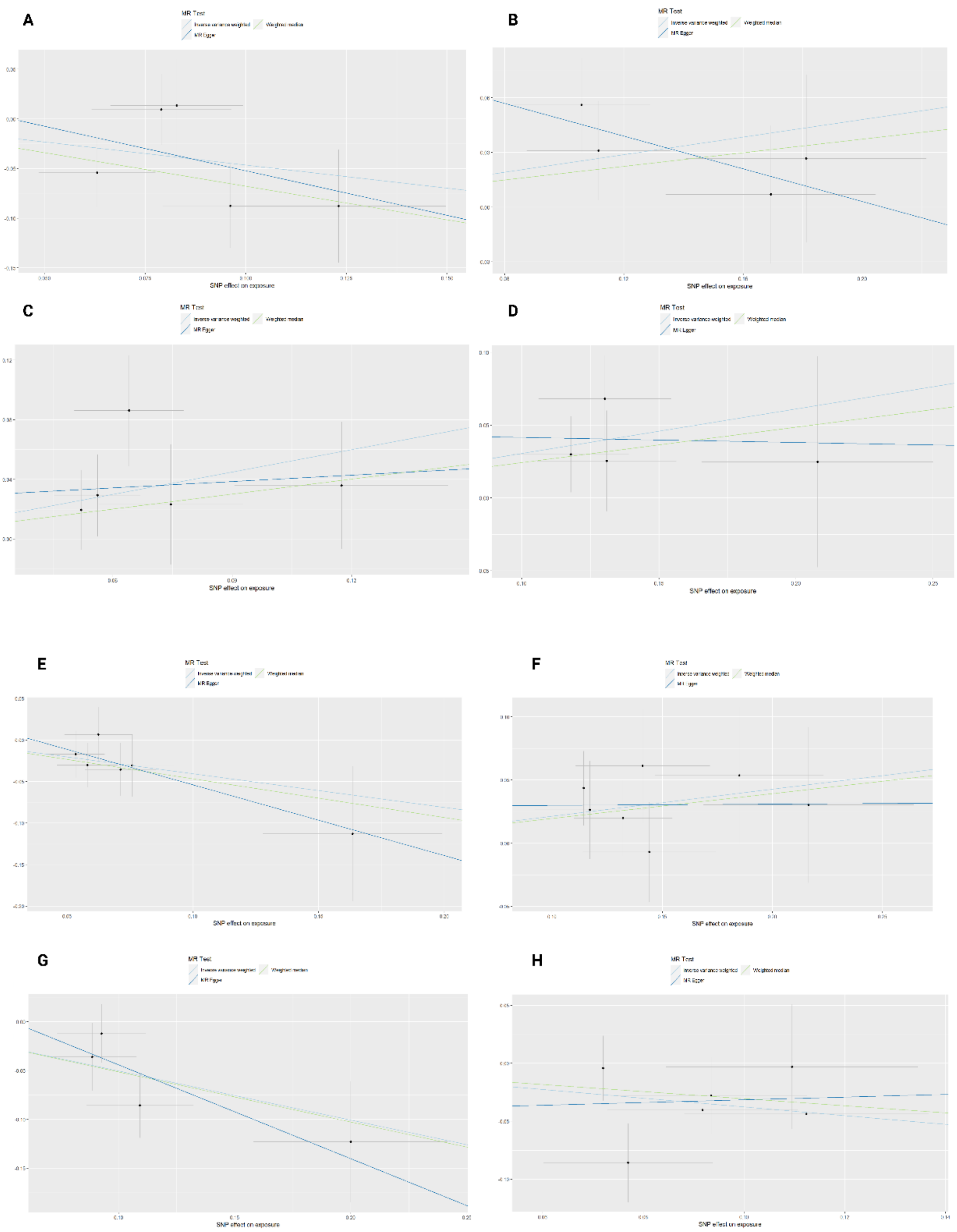

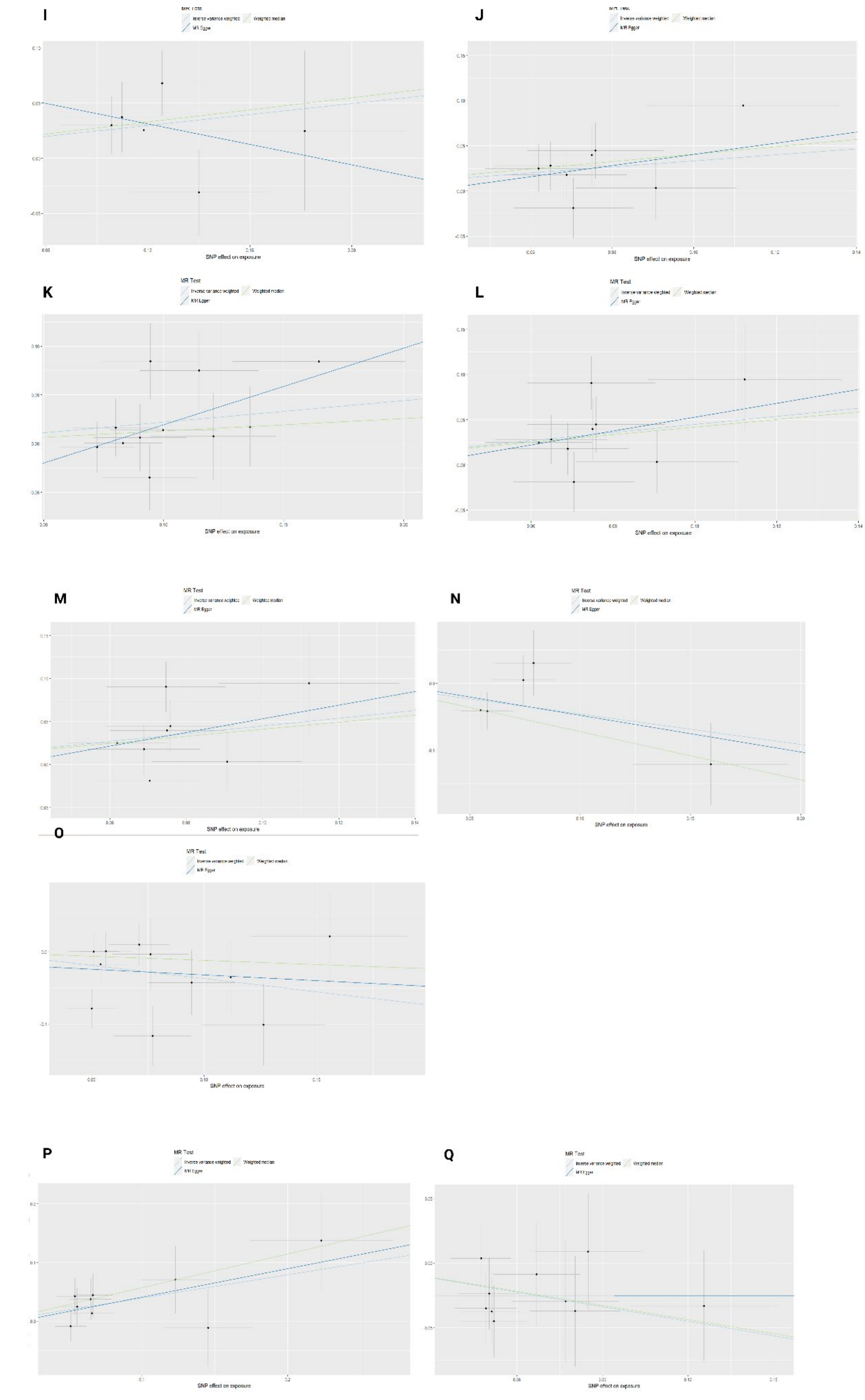

Supplement Figure S2

Funnel plots of significant and nominal significant from GM on DN. (A: order.Rhodospirillales;B: order.Verrucomicrobiales; C: phylum.Bacteroidetes;D: phylum.Proteobacteria;E: class. Actinobacteria; F:class.Verrucomicrobiae, SNP>8 can be drawn)

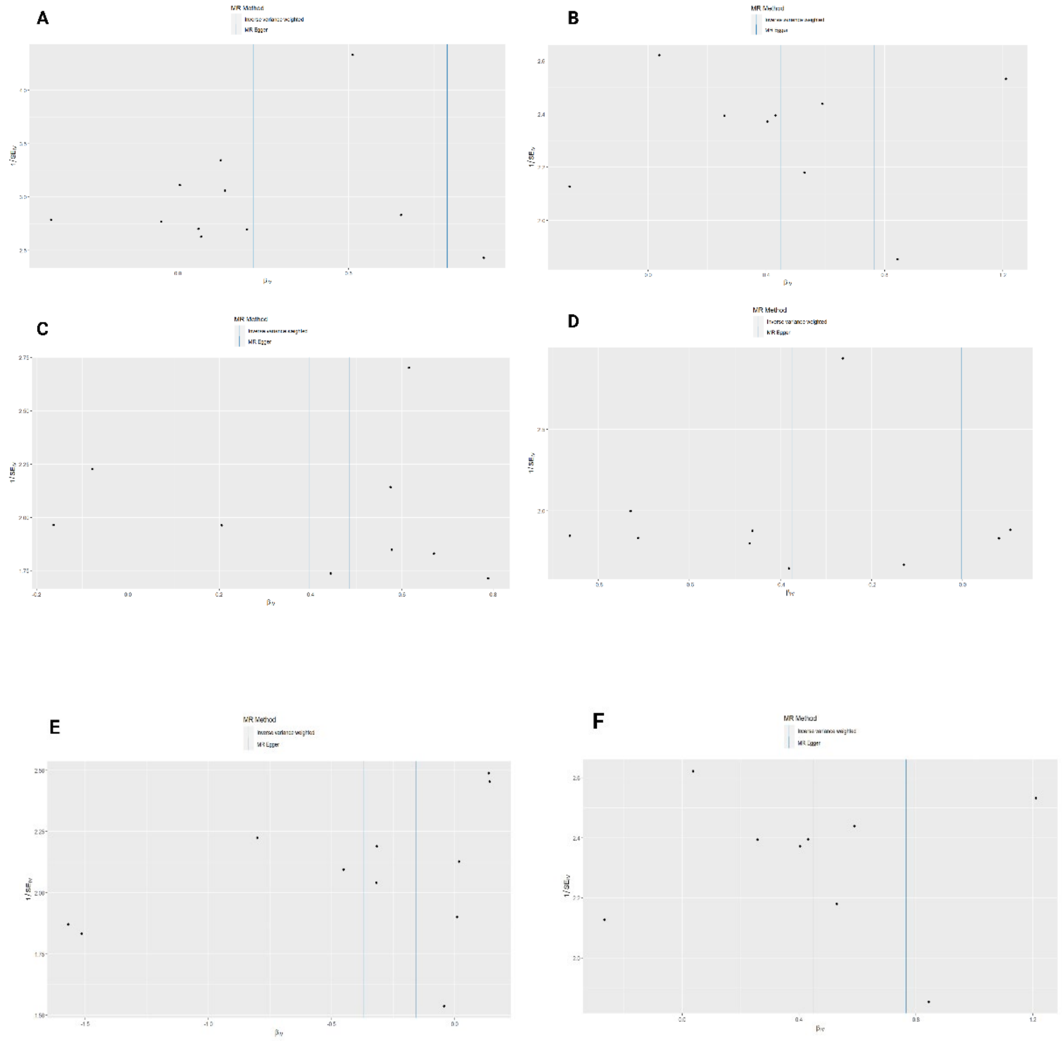

Supplement Figure S3

Leave-one-out plots of significant and nominal significant from GM on DN. (A: order.Rhodospirillales; B: order.Verrucomicrobiales; C: phylum.Bacteroidetes; D: phylum.Proteobacteria; E: class. Actinobacteria; F:class.Verrucomicrobiae; G:family.Verrucomicrobiaceae; H:genus.Tyzzerrlla3; I:genus.Oxalobacter J:genus.Lachnoclostridium; K:genus.RuminococcaceaeUCG002; L:genus.Howardella; M:genus. Eubacteriumcoprostanoligenesgroup; N:family.Oxalobacteraceae; O:class.Gammaproteobacteria)

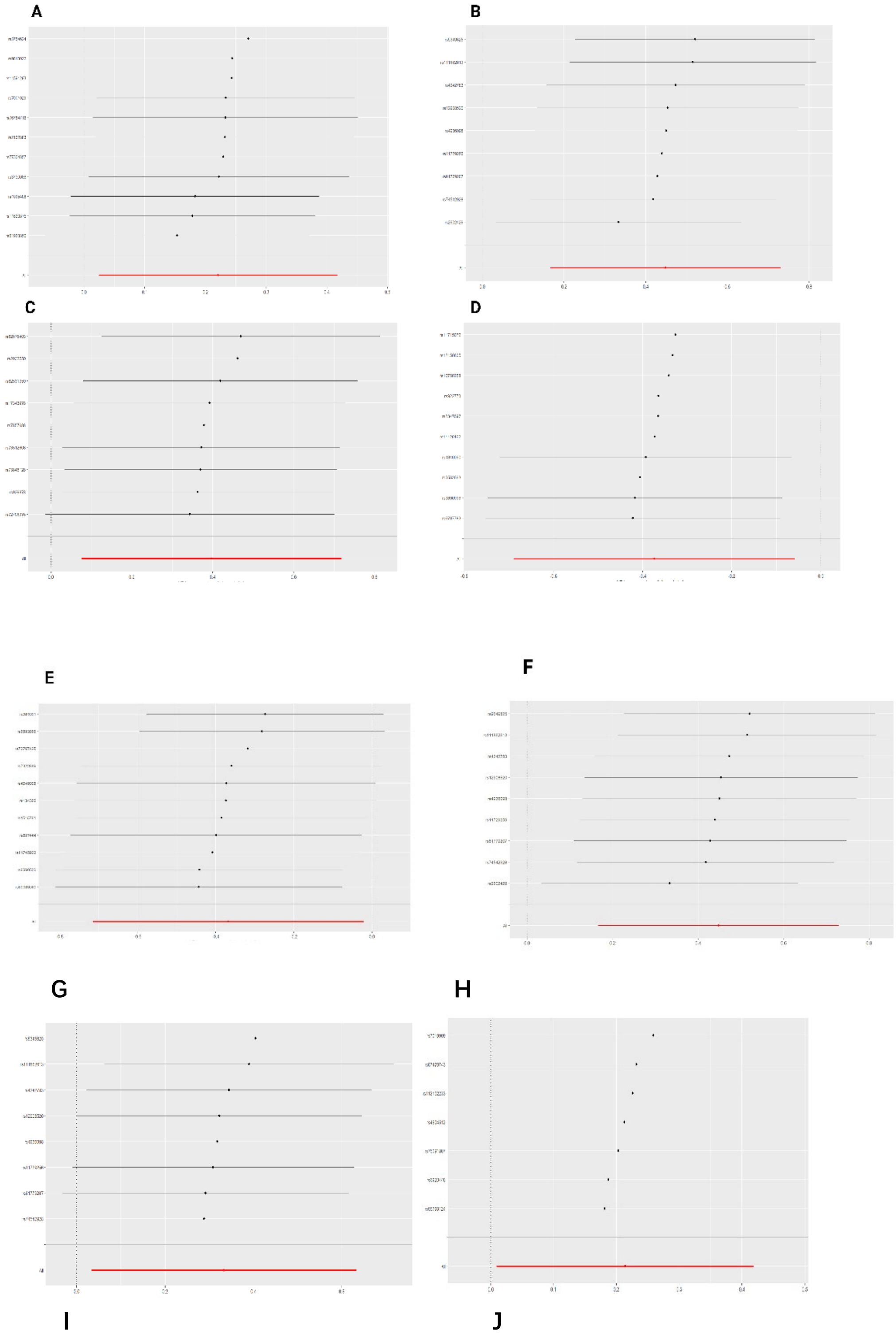
A
B
C
D
E
F
G
H
I
J

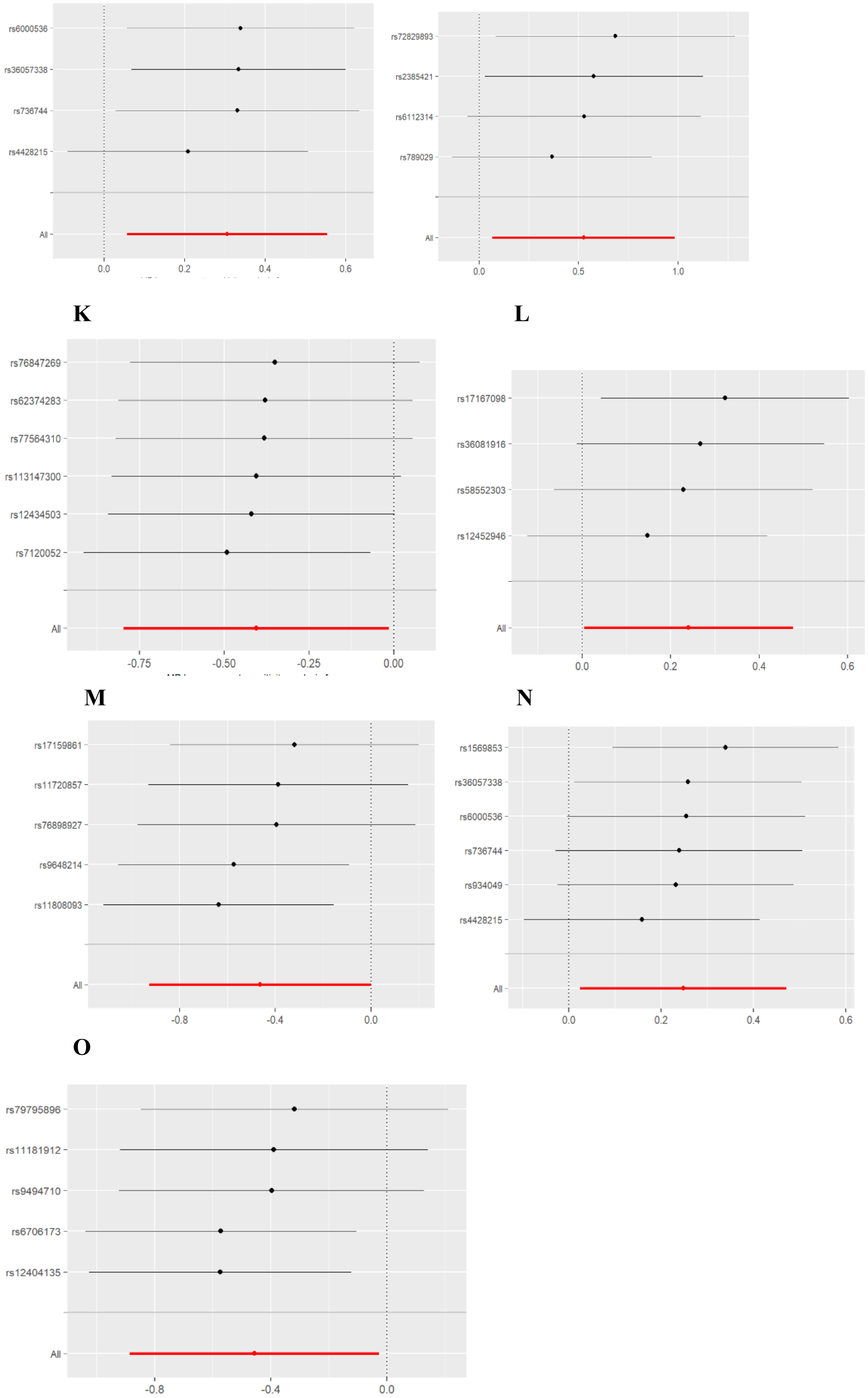

Supplement Figure S4

Scatter plots of reverse MR analysis between DN and causal GM

(A:order.Rhodospirillales; B:phylum.Proteobacteria, IVW: P<0.05).

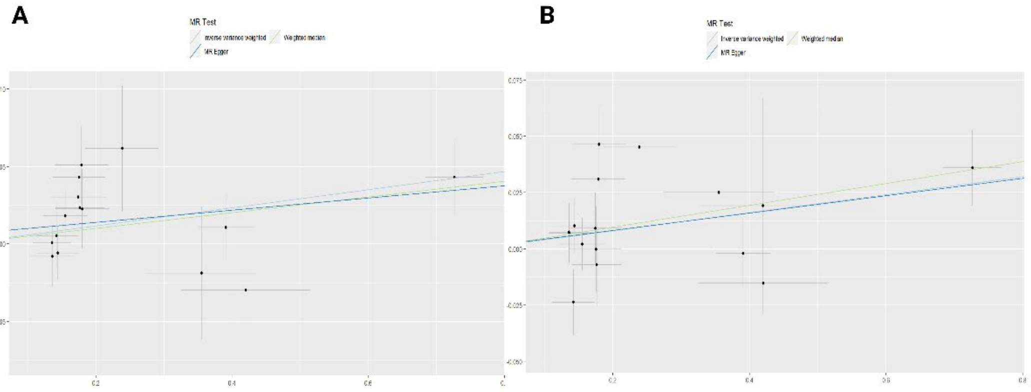

Supplement Figure S5

Funnel plots of reverse MR analysis between DN and causal GM (A:order.Rhodospirillales; B:phylum.Proteobacteria, IVW: P<0.05).

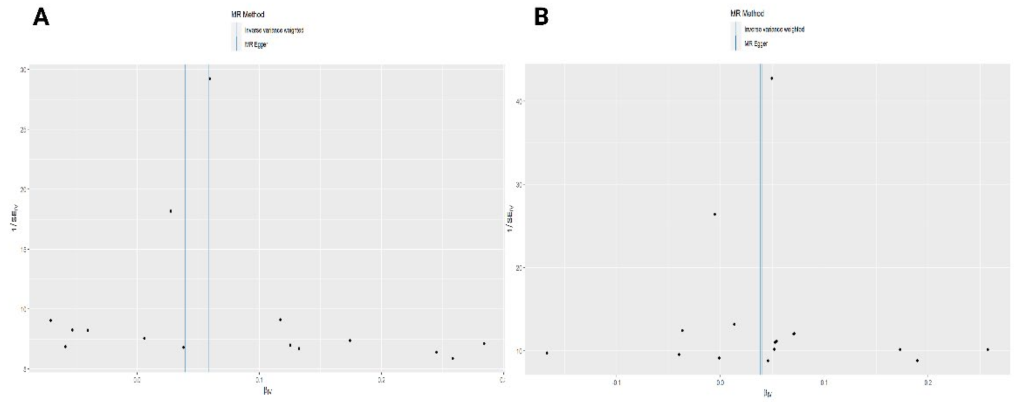

Supplement Figure S6

Leave-one-out plots of reverse MR analysis between DN and causal GM (A:order.Rhodospirillales; B:phylum.Proteobacteria, IVW: P<0.05).

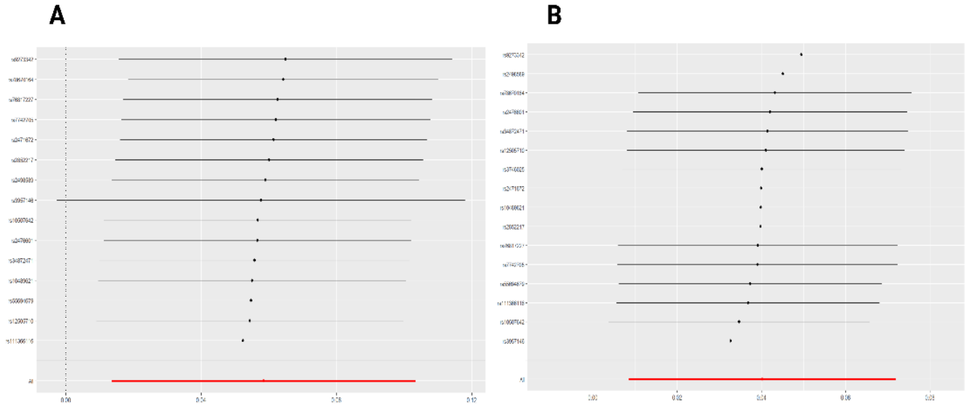

Supplement Figure S7

Scatter plots of MR analysis between T2DN and 19 causal GM. (A: family.Verrucomicrobiaceae;B:order.Rhodospirillales;C:order.Verrucomicrobiales; D:class.Verrucomicrobiaea, IVW: P<0.05)

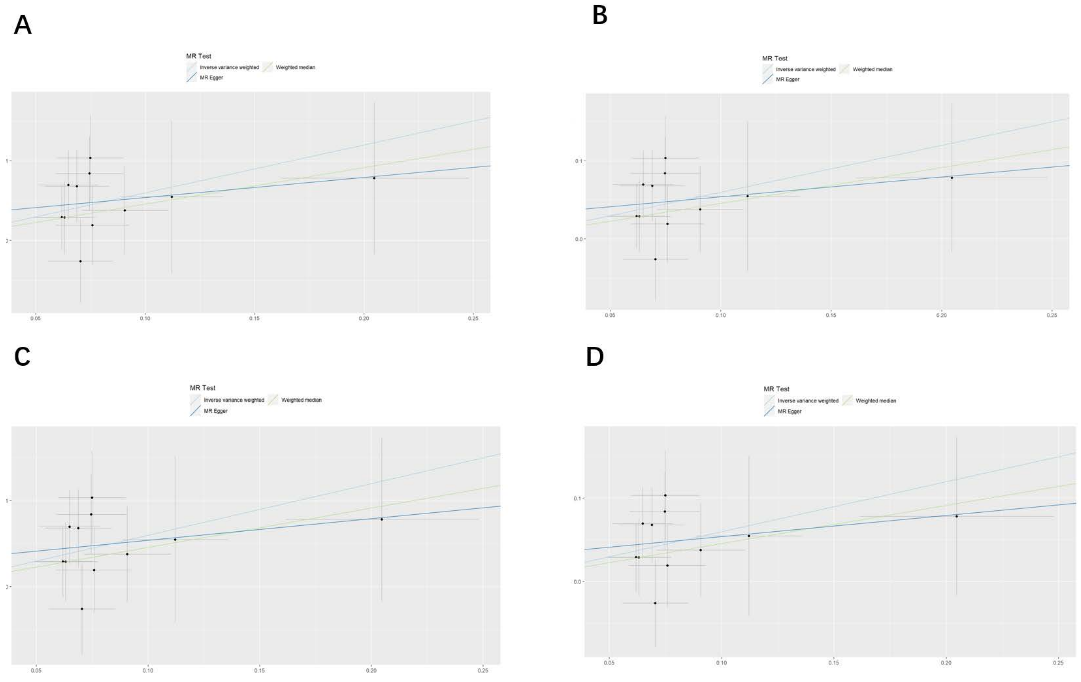

Supplement Figure S8

Funnel plots of MR analysis between T2DN and 19 causal GM.

(A: family. Verrucomicrobiaceae;
B:order.Rhodospirillales;C:order.Verrucomicrobiales;
D:class.Verrucomicrobiaea, IVW: P<0.05)

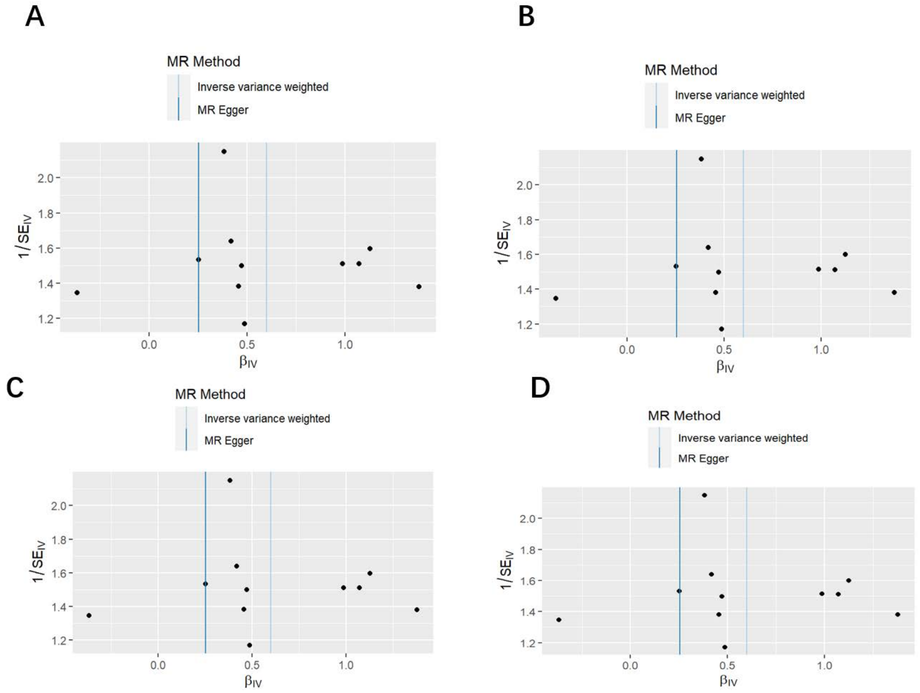

Supplement Figure S9

Leave-one-out plots of MR analysis between T2DN and 19 causal GM.
(A: family. Verrucomicrobiaceae;
B:order.Rhodospirillales;C:order.Verrucomicrobiales;
D:class.Verrucomicrobiaea, IVW: P<0.05)

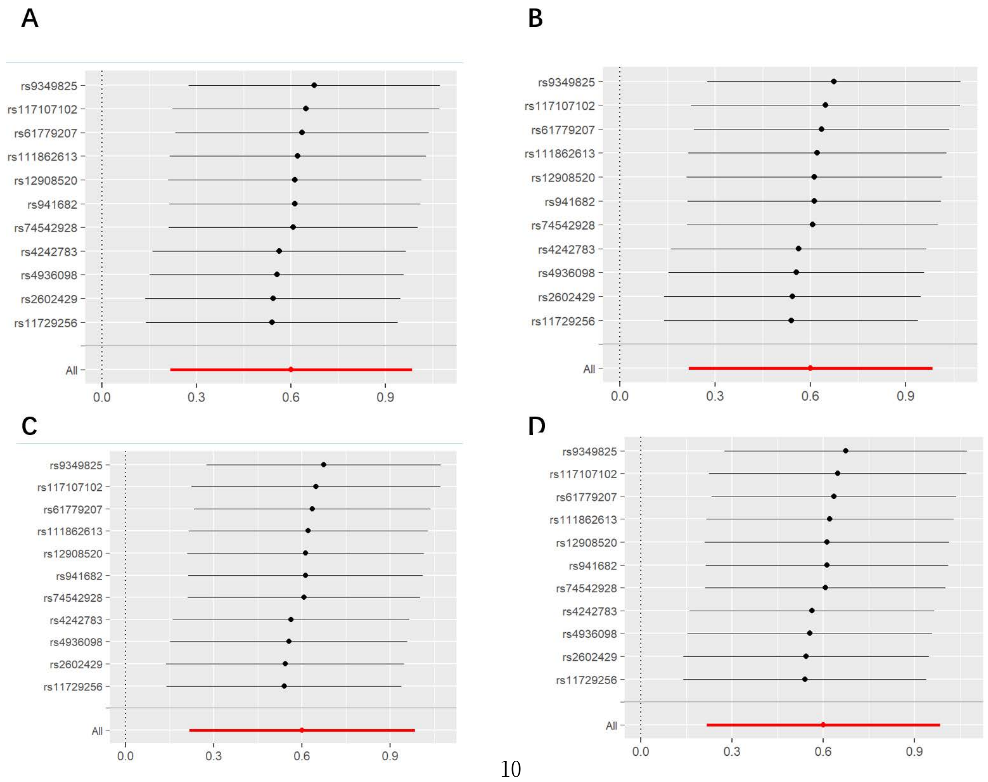

Supplement Figure S10

MR analysis between T1DN and Genus. Eubacerium.

A: Scatter plots of MR analysis

B: Funnel plots of MR analysis

C: Leave-one-out plots of MR analysis

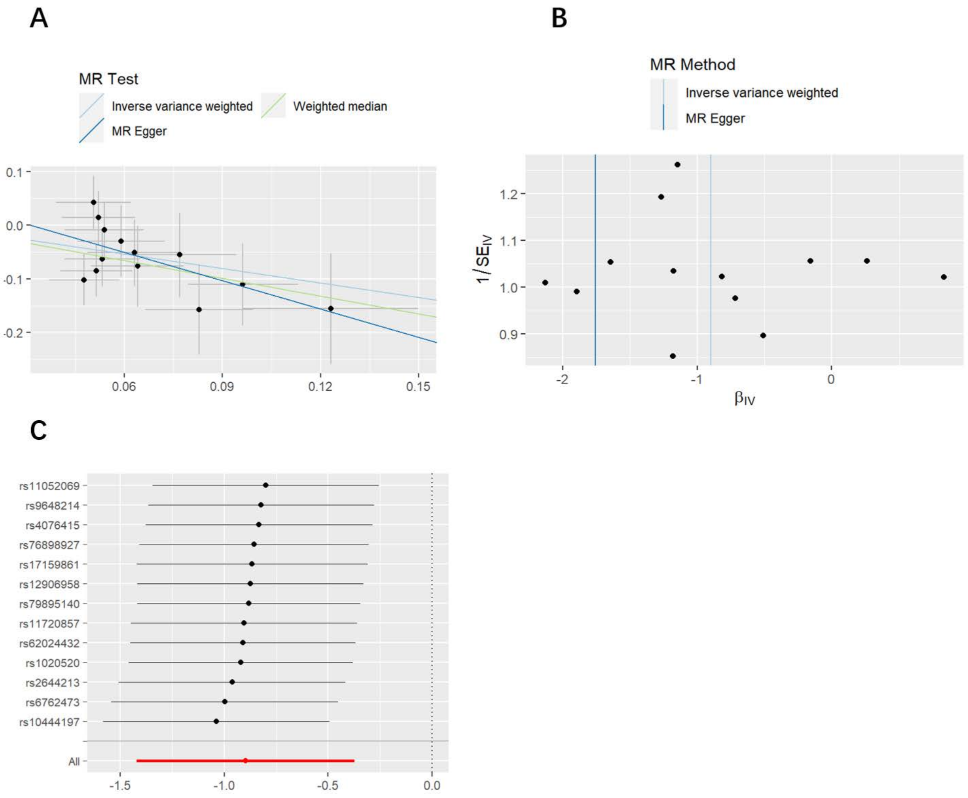

Supplement: Supplementary file 1 [file DataSheet_1.pdf]
